# Development of a Matrix‐Assisted Laser Desorption Ionization High Resolution Mass Spectrometry Method for the Quantification of Camalexin and Scopoletin in *Arabidopsis thaliana*

**DOI:** 10.1002/rcm.9973

**Published:** 2024-12-18

**Authors:** Leonardo Parasecolo, Ivan M. Monsalvo, Nikola Kovinich, Demian R. Ifa

**Affiliations:** ^1^ Department of Chemistry, Faculty of Science York University Toronto Ontario Canada; ^2^ Department of Biology, Faculty of Science York University Toronto Ontario Canada

**Keywords:** Mass spectrometry, Method development, Phytoalexin, plant biology

## Abstract

**Rationale:**

Understanding plant defense mechanisms against pathogens is essential for enhancing agricultural productivity and crop protection. This study focuses on the quantification of camalexin and scopoletin, two critical phytoalexins in *Arabidopsis thaliana*, using mass spectrometry techniques. Precise measurement of these compounds provides insights into plant resistance and supports agricultural research.

**Methods:**

Camalexin and scopoletin were quantified using matrix‐assisted laser desorption ionization high‐resolution mass spectrometry (MALDI‐HRMS). The matrix and solvent conditions were optimized to maximize sensitivity and accuracy. MS/MS experiments confirmed compound identification with high mass accuracy (mass error < 5 ppm). The method was validated through comparative analysis of wild‐type (WT) and mutant *Arabidopsis* lines, using internal standards and multiple replicates to ensure precision and reliability.

**Results:**

The method exhibited high linearity for scopoletin (*R* ^2^ = 0.9992) and camalexin (*R* ^2^ = 0.9987) across concentration ranges of 0.16–5 and 0.31–5 μM, respectively. Limits of detection (LOD) were 0.16 μM for camalexin and 0.04 μM for scopoletin, with limits of quantification (LOQ) at 0.2 μM and 0.08 μM, respectively. Samples analysis demonstrated reliable quantification in WT and mutant lines, with significant reductions in camalexin and scopoletin levels observed in the *atwrky33‐2* and *atmyb15‐1* mutants, respectively. Additionally, the method detected sub‐physiological concentrations, confirming its sensitivity and robustness for low‐level detection.

**Conclusions:**

This study presents a validated, precise, and accurate MALDI‐HRMS method for the quantification of camalexin and scopoletin in *Arabidopsis thaliana*. The approach not only enhances understanding of plant defense mechanisms but also offers potential applications for biotechnological and agricultural research, especially for investigating genetic variations and stress‐induced phytoalexin production.

## 1 Introduction

Phytoalexins are specialized low‐molecular‐weight metabolites produced by plants in response to pathogen attacks. They vary in structure among plant species [1, 2] and are crucial for agricultural resistance to pathogens [1, 2]. For example, glyceollins confer resistance in soybean to *Phytophthora sojae* and *Macrophomina phaseolina* [3, 4], while camalexin provides resistance in *Arabidopsis thaliana* to *Plasmodiophora brassicae* and *Alternaria brassicicola* [3, 4]. For the last three decades, *Arabidopsis thaliana* has served as a model to understand how the production of phytoalexins is regulated since it can produce at least 12 different phytoalexins in response to biotic stressors [1]. The most well studied are camalexin and scopoletin, for which all the enzymes involved in their biosynthesis have been characterized [1]. Camalexin is an indole alkaloid that is involved in providing resistance to *Botrytis cinerea*, *Piriformospora indica*, *Alternaria brassicicola*, and *Pseudomonas syringae* [5]. Similarly, scopoletin is a phytoalexin that provides resistance to various pathogens including bacteria, fungi, and protozoa [6]. Numerous studies highlight the genes involved in phytoalexin synthesis, underscoring their significance in plant biology [7, 8, 9]. Beyond agriculture, phytoalexins also show potential for treating human diseases [10, 11, 12].

MS is crucial for plant metabolomics, offering comprehensive insights into plant metabolomes, metabolism, and interactions with pathogens [13]. It has advanced our understanding of plant adaptation to environmental and biotic stresses [14]. Despite challenges in identifying unknown metabolites and accurate quantitative analysis, ongoing MS advancements show promise [15].

Analytical methods for the quantification of camalexin and scopoletin have been developed over the past decades [16, 17, 18, 19, 20, 21, 22, 23, 24]. While these methods demonstrate remarkable analytical performance, particularly in sensitivity, they often require extensive sample preparation and significant solvent use, leading to high costs and prolonged analysis times. These factors pose considerable drawbacks, especially when processing large sets of samples.

MALDI‐MS outstands for biomolecule analysis due to its speed and ease of use. Initially used for qualitative analysis, it now supports quantitative analysis despite challenges with low‐mass analytes [25]. Optimizing experimental parameters and sample preparation, especially matrix selection and crystallization, minimizes variability. Careful design and optimization allow for meaningful quantitative comparisons, making MALDI‐MS valuable in biological research [25, 26].

This study developed a MALDI‐HRMS method for the absolute quantification of camalexin and scopoletin in *Arabidopsis* plants: The method's performance was evaluated for linearity, LOD, LOQ, accuracy, and precision to ensure reliability and reproducibility. This advancement provides a rapid, precise, and accurate tool for studying plant defense mechanisms and supports future research in plant biology, biotechnology, agriculture, and pharmaceuticals.

## 2 Experimental Methods

### 2.1 Reagents

All reagents used in this study were sourced from Sigma‐Aldrich (Oakville, Canada), unless otherwise stated. These included Murashige and Skoog (MS) medium with vitamins, sucrose (≥ 99.5%), and Gelzan CM for seed plating and media preparation. Solvents for extraction included methanol (HPLC grade), water (LC–MS grade), acetic acid (≥ 99.7%), acetone (≥ 99.5%), and acetonitrile (HPLC‐MS grade). MALDI matrices—9‐aminoacridine (9‐AA, ≥ 99.5%), 5‐chloro‐2‐mercaptobenzothiazole (CMBT, ≥ 90%), and 1,5‐diaminonaphthalene (DAN, ≥ 95%)—were used for ionization studies. Ethanol (≥ 99.5%) and methanol (HPLC grade) were employed to extract *Taraxacum officinale* powder. Standards included scopoletin (≥ 99%) and camalexin (≥ 98%), with indolepropionic acid (≥ 99%) and daidzein (≥ 98%) as internal standards. Liquid nitrogen was used for snap‐freezing samples. The synthetic elicitor flg22 peptide was purchased from PhytoTech Lab. Pierce Negative Ion Calibration Solution for negative ionization mode was purchased from Thermo fisher (Thermo Fisher Scientific, San Jose, CA).

### 2.2 Plant Metabolite Extraction

For method optimization and target compound detection, *Arabidopsis* seeds were sterilized following Denoux et al. [27]. The sterilized seeds were plated on solid MS medium with vitamins, along with 1% sucrose and 2.5 g/L Gelzan to solidify the medium, at pH 5.8. Seeds were kept in darkness at 4°C for 3 days, exposed to cool white T5 fluorescent lights (100 μEm^2^/s) for 5–6 h, and returned to darkness at 22°C for 3 days. Seedlings were transfer into a 16‐h photoperiod using cool white T5 fluorescent lights (100 μEm^2^/s). Five‐day‐old seedlings (15–20) were transferred to liquid MS medium in 12‐well plates and grown for five more days. A day before treatment, the medium was replaced with fresh MS medium. On day 10, the seedlings were treated with 5‐μM flg22 (a 22‐amino acid peptide fragment from bacterial flagellin that triggers phytoalexin production; QRLSTGSRINSAKDDAAGLQIA) for 16 h. They were then snap‐frozen in liquid nitrogen, lyophilized, weighed, and homogenized using a ball mill [27]. For extraction, 1 mL of methanol was added to the homogenized sample, followed by 30 min of sonication and centrifugation at 13 200 *g* for 15 min. The supernatant was collected and dried under nitrogen gas (first extract). A second extraction was performed using 20 μL of MeOH:H2O:AcOH (80:19:1) per mg of dry sample. The supernatant from this step was used to dissolve the first extract for subsequent MS analysis.

### 2.3 MALDI‐HRMS Condition Optimization

MALDI‐HRMS experiments were conducted using a Q‐Exactive mass spectrometer (Thermo Fisher Scientific) equipped with a Spectroglyph ESI/MALDI ion source (Spectroglyph LLC, Kennewick, WA). The acquisitions were performed with a resolving power of 70 000 at *m*/*z* 200, a maximum injection time of 200 ms, AGC target = 1E6, and an *m*/*z* range of 100–1000. The acquired ion intensities were extracted using Thermo Xcalibur Qual Browser. To optimize camalexin detection, various MALDI matrices known for ionizing small molecules [28, 29] were tested: 9‐AA and CMBT in negative mode and DAN in positive and negative modes. These matrices were solubilized until saturation and evaluated with different solvents (acetone, methanol, and acetonitrile). Plant extract and matrix solutions were mixed 1:1, and 4 μL of the mixture was applied to a Teflon‐coated slide (Tekdon Incorporated, Myakka City, FL), dried, and analyzed. Laser currents were set to 2 A for CMBT, 1.8 A for DAN, and 2.5 A for 9‐AA, with a repetition rate of 300 Hz. Each acquisition was performed by scanning the sample for 30 s.

The identification of target compounds, scopoletin (*m*/*z* 191.0350) and camalexin (*m*/*z* 199.0330), involved analyzing standard solutions and comparing them with treated samples. Only *m*/*z* values within 5 ppm of the exact target masses were considered. Fragmentation spectra from both standard solutions and flg22‐treated samples were cross‐referenced with the Human Metabolome Database (HMDB) for further confirmation [30]. MS/MS analysis used a normalized collision energy of 40.0 (*z* = 1) with a laser current of 1.8 A, a 300‐Hz repetition rate, and an acquisition range of *m*/*z* 50–200. Target ions were monitored at *m*/*z* 191.0 ± 0.2 for scopoletin and 199.0 ± 0.2 for camalexin (Figure S1).

### 2.4 Preparation of Samples for Calibration and Analysis

A biological matrix resembling the *Arabidopsis* metabolome was created as follows: *Taraxacum officinale* samples were washed with ethanol, dried at 60°C, and ground into a powder. Metabolites were extracted using 80% methanol at 20 μL per mg of powder. The mixture was centrifuged (5 min at 14 K RPM) to separate the supernatant, which was then divided into 40‐μL aliquots. These aliquots were used to create calibration curves and quality controls at low, medium, and high concentrations.

### 2.5 Blank Analysis

Before adding the target analytes and their internal standards to the biological matrix, one portion was combined with a matrix solution in a 1:1 ratio. This mixture was then analyzed using MALDI‐HRMS to confirm the absence of target compounds, internal standards, or any other isobar substances derived from either the MALDI matrix or the biological matrix (Figures S2 and S3). The blank analysis was conducted in triplicate alongside the calibration curve and quality control analysis to strengthen the reliability of our findings.

### 2.6 Standard Addition, Internal Standard Selection, and Calibration Curve

**FIGURE 1:**
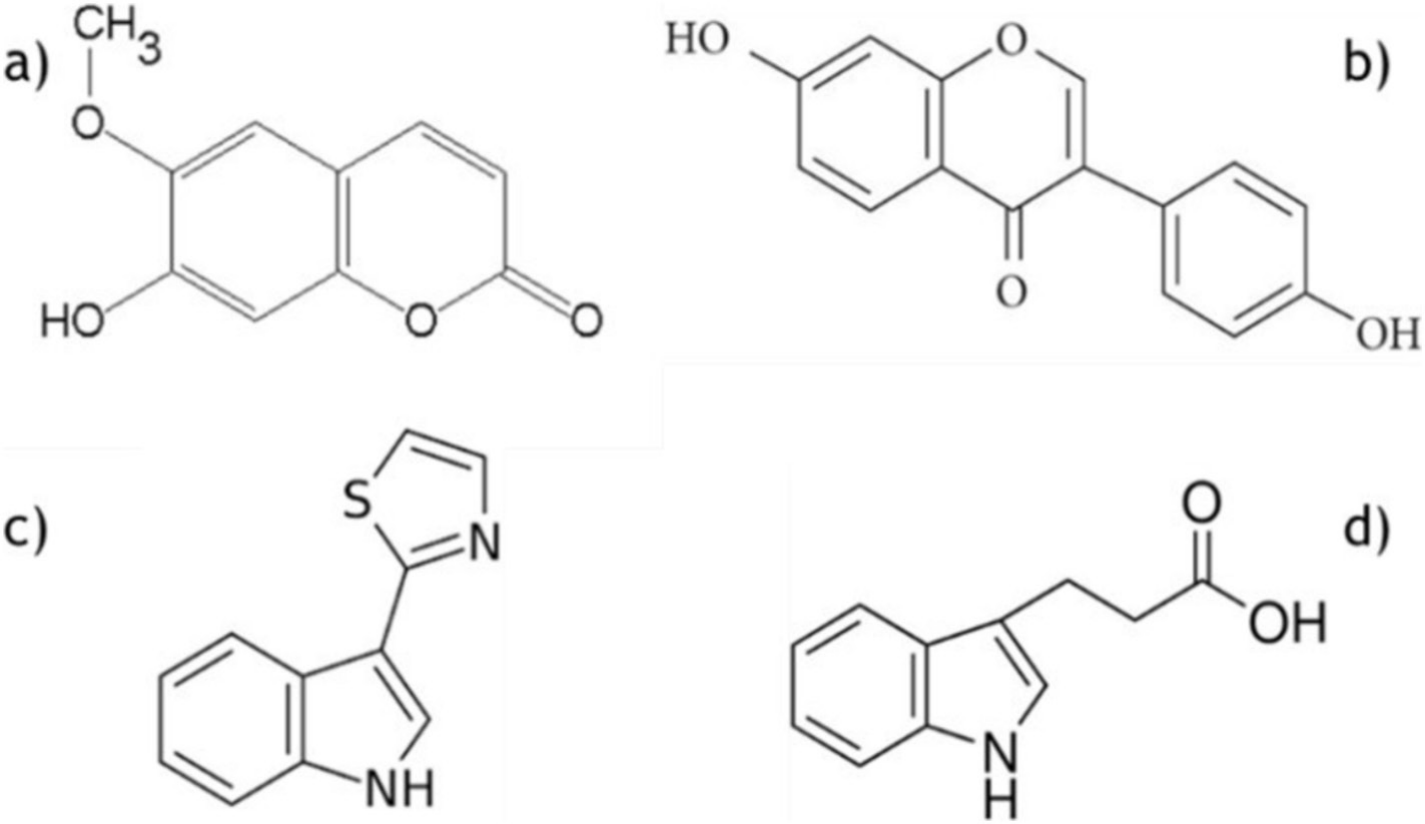
Target analytes and their internal standards: (a) scopoletin, (b) daidzein, (c) camalexin, and (d) 3‐indole‐propionic acid.

After confirming the absence of interference, 40‐μL aliquots were spiked with 5 μL of camalexin and scopoletin standards (0.39–50 μM), yielding final concentrations of 0.04–5 μM (0.16–20 μg/g for camalexin, 0.16–19.20 μg/g for scopoletin) after adding 5 μL of internal standards (IS). These concentrations align with literature values for elicited *Arabidopsis* [16, 17, 18, 19, 20, 21, 22, 23, 24]. Indolepropionic acid (1.25 μM) and daidzein (0.62 μM) were used as IS for camalexin and scopoletin (Figure 1), respectively, with IS concentrations set at the calibration curve midpoint. Each calibration curve point was acquired in duplicate (*n* = 2), according to and exceeding FDA guidelines, which typically require only single replicates (*n* = 1).

### 2.7 Evaluation of Accuracy, Precision, and Sensitivity

The accuracy and precision of the method were assessed through triplicate analyses of quality control samples prepared at three different concentrations: 4 μM (QCH), 1 μM (for camalexin), and 0.9 μM (for scopoletin) (QCM), as well as 0.5 μM (for camalexin) and 0.45 μM (for scopoletin) (QCL). Additionally, the limits of detection (LOD) and quantification (LOQ) were determined by preparing batches at decreasing concentrations until a signal‐to‐noise ratio of at least 3 and 10, respectively, was achieved.

### 2.8 *Arabidopsis thaliana* Samples Analysis

The developed method was applied to the analysis of Arabidopsis thaliana extracts, prepared as described in Section 2.2. Quantitative comparisons were conducted between wild‐type (WT) and mutant lines for each compound. For camalexin quantification, we compared the WT with the *atwrky33‐2* mutant, which is expected to exhibit reduced camalexin levels [8]. Similarly, for scopoletin quantification, the WT was compared to the *atmyb15‐1* mutant, anticipated to show diminished scopoletin production [31]. WT and both mutant lines were obtained from the Arabidopsis Biological Resource Center (ABRC).

Each line underwent two treatments: flg22 (an elicitor) and DMSO (negative control), resulting in six sample groups. Each group consisted of three biological replicates, and each biological replicate was analyzed in triplicate to ensure precision and minimize analytical variability.

For sample preparation, after metabolite extraction (Section 2.2), 10 μL of internal standard solution was added to 40 μL of the extracted metabolome. Ten microliters of indolepropionic acid (6.25 μM) was added to both *atwrky33‐2* mutant and WT samples for camalexin quantification, and 10 μL of daidzein (3.1 μM) was added to both *atmyb15‐1* mutant and WT samples for scopoletin quantification. Then, 20 μL of each prepared sample was mixed with 20 μL of saturated DAN‐acetone solution, and 4 μL of the mixture was aliquoted onto a Teflon slide, dried, and analyzed following the developed method. The final concentrations of indolepropionic acid (1.25 μM) and daidzein (0.62 μM) were consistent with the calibration curve and QC samples, ensuring accurate quantification of camalexin and scopoletin, respectively.

## 3 Results and Discussion

The analytical performance of the method for absolute quantification of camalexin and scopoletin was comprehensively evaluated.

### 3.1 Matrix Selection

**FIGURE 2:**
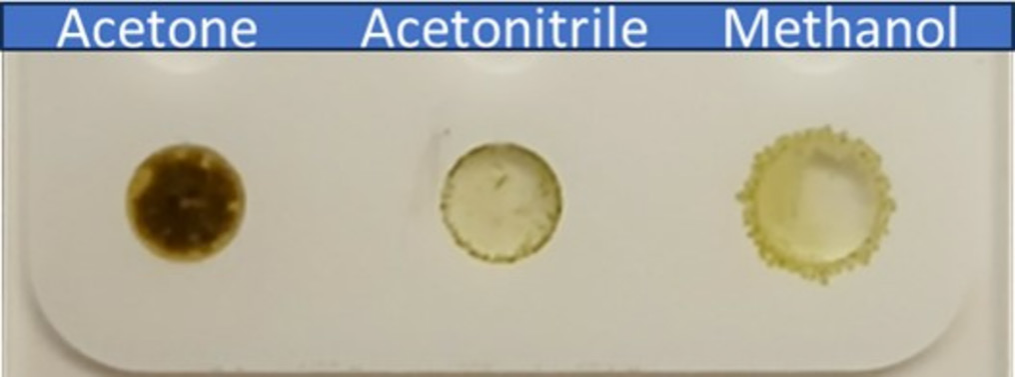
Acetone facilitated superior solubilization of DAN, resulting in greater deposition of the MALDI matrix on the slide. Its rapid evaporation promoted more uniform crystallization. These factors likely contributed to the enhanced signal intensity observed, making acetone the preferred solvent for method development.

Initial tests revealed low or no signals with 9‐AA and CMBT in negative mode and DAN in positive ion mode. In contrast, the saturated solutions of DAN in methanol or acetone, in negative mode, produced promising results. These combinations (DAN + acetone and DAN + methanol) were evaluated in triplicate and compared (Table 1). Acetone's rapid evaporation and superior solubilization of DAN promoted consistent crystallization, improving signal strength and reproducibility, establishing it as the optimal solvent for method development (Figure 2).

**TABLE 1:** Ion counts for the *m*/*z* values corresponding to scopoletin and camalexin, emphasizing DAN combined with acetone's superior performance.

	Replicate 1	Replicate 2	Replicate 3	Average
Scopoletin				
Methanol + DAN	6.43E2	2.51E2	3.73E2	4.22E2
Acetone + DAN	1.31E3	2.18E3	1.88E3	1.79E3
Camalexin				
Methanol + DAN	2.78E2	9.02E2	7.84E2	6.54E2
Acetone + DAN	2.28E3	9.44E3	2.47E3	4.73E3

*Note:* Variability is noted, stemming from the decision to omit normalization in this phase of the study, which may have been unnecessary.

### 3.2 Calibration Curve and Linearity

**FIGURE 3:**
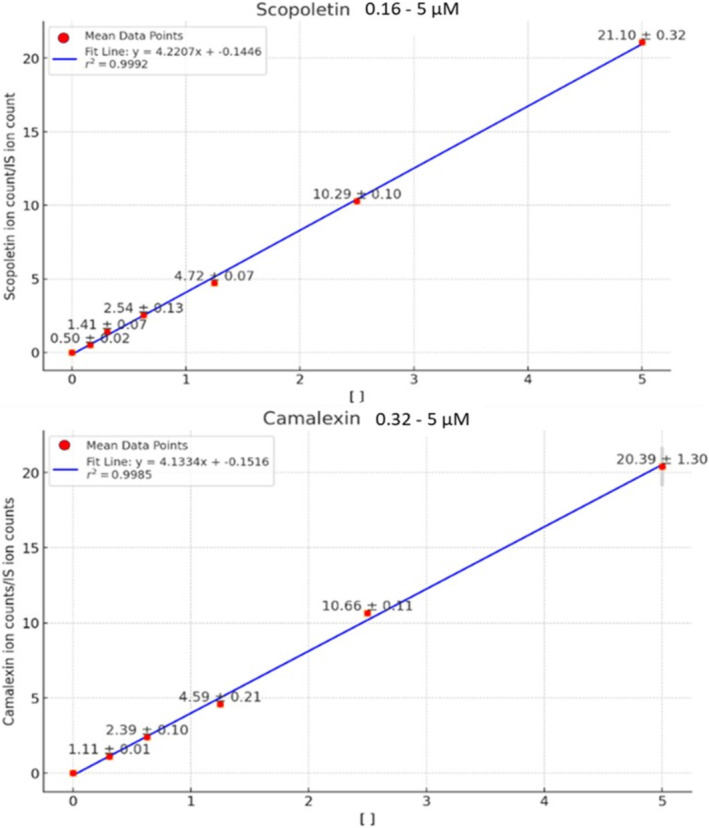
Calibration curve assessed for scopoletin (top) and camalexin (bottom). On the X axis is reported the range of spiked concentration and on the Y axis the signal of the target compound divided by the signal of its internal standard.

For camalexin and scopoletin, the concentration ranges for the calibration curves were determined based on achieving a signal‐to‐noise ratio (S/N) exceeding 10. Consequently, a concentration range of 0.16–5 μM (0.64–19.20 μg/g) was established for scopoletin, while a range of 0.31–5 μM (1.25–20 μg/g) was chosen for camalexin. The linearity of the method was verified by examining the obtained *R* ^2^ values, both exceeding 0.99. Each point was analyzed in duplicate and the reported results for each point are the average values obtained by the replicate analyses (Figure 3).

### 3.3 Sensitivity Assessment

The sensitivity of the method was evaluated by determining the LOD and LOQ for camalexin and scopoletin. Camalexin's LOD was established at a concentration of 0.16 μM, with an S/N of 4.68, while scopoletin's LOD was assessed at 0.04 μM, yielding a S/N ratio of 4.24. LOQ for camalexin was determined to be 0.2 μM, with an S/N of 10.95, and for scopoletin, it was 0.08 μM, with an S/N of 10.90 (Figure S4).

### 3.4 Accuracy and Precision Assessment

Accuracy and precision were evaluated by analyzing quality control (QC) samples in triplicate. Satisfactory performance was observed across all concentration levels for both camalexin and scopoletin. Notably, optimal outcomes were achieved for QC samples at medium concentrations, indicative of typical sample compositions. Table 2 summarizes the accuracy, expressed as relative error, and variability, expressed as the coefficient of variability, for camalexin and scopoletin QC samples at different concentration levels. The method demonstrates sufficient sensitivity for detecting and quantifying camalexin across various samples, including WT and mutated varieties. Moreover, it exhibits exceptional sensitivity for scopoletin, enabling detection even at sub‐physiological concentrations.

**TABLE 2:** Accuracy, expressed as relative error (RE), and variability, expressed as relative standard deviation (RSD), are shown for camalexin and scopoletin.

		RE	RSD
Camalexin	QC high 4 μM	6.03%	3.25%
	QC medium 1 μM	1.14%	0.15%
	QC low 0.5 μM	−4.55%	0.90%
Scopoletin	QC high 4 μM	0.57%	3.99%
	QC medium 0.9 μM	−1.96%	1.10%
	QC low 0.45 μM	−9.79%	2.30%

*Note:* For each compound, the values obtained for QC samples prepared at high (*n* = 3), medium (*n* = 3), and low (*n* = 3) concentrations.

### 3.5 *Arabidopsis thaliana* Samples Analysis

**FIGURE 4:**
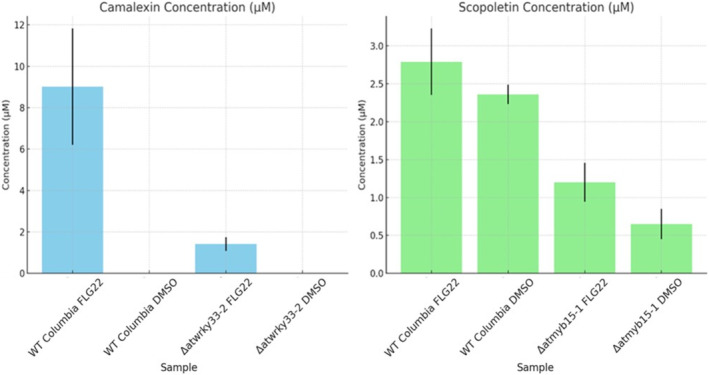
Left, concentrations of camalexin in WT FLG22, WT DMSO, mutated *atwrky33‐2* FLG22, and mutated *atwrky33‐2* DMSO. Right, concentrations of scopoletin in WT FLG22, WT DMSO, mutated *atmyb15‐1* FLG22, and mutated *atmyb15‐1* DMSO. Error bars are representative of biological variability.

The results of the sample analysis, presented in Figure 4, show that concentrations in WT samples align with values reported in previous studies using various analytical methods [16, 17, 18, 19, 20, 21, 22, 23, 24]. The method demonstrates high reliability, accurately detecting both physiological and sub‐physiological concentrations, highlighting its sensitivity to genetic variation. Significant reductions in camalexin levels were observed in the *atwrky33‐2* mutants [8], while *atmyb15‐1* mutants exhibited reduced scopoletin levels [31].

It is worth noting that in one biological replicate of the *atwrky33‐2* mutant, despite achieving a signal‐to‐noise ratio above 10, the calculated concentration (0.24 μM, Table S1) fell below the lowest point of the calibration range. Nevertheless, camalexin was still detected, demonstrating that the method is sensitive enough to identify trace amounts, even when they fall outside the validated calibration range, ensuring reliable detection of low‐level presence.

The method's specificity is further confirmed by camalexin being below the LOD in non‐elicited samples, consistent with its inducible response to biological stress [24, 27]. In contrast, scopoletin was detected in both DMSO‐ and flg22‐treated samples, supporting previous reports that it is present under normal physiological conditions as its glucosinolate form and hydrolyzed upon abiotic stress [31, 32, 33].

In WT samples, scopoletin concentrations were predominantly found in the low‐to‐mid range of the calibration curve. However, the higher end of the calibration range may be useful for quantifying scopoletin under conditions of enhanced production, such as elicitation by abiotic stress [33] or in genetically modified organisms where its biosynthesis is upregulated. These findings validate the capability of the method for comparing WT and mutant varieties under both basal and stress‐induced conditions.

## 4 Conclusion and Future Directions

We have developed a method for the absolute quantification of camalexin and scopoletin, two crucial phytoalexins in *Arabidopsis*, using MALDI‐HRMS. Our optimization of matrix and solvent conditions, along with method validation, showed strong linearity, sensitivity, accuracy, and precision, even at low concentrations. The method was validated using *Arabidopsis* wild‐type (WT) plants and mutant lines, specifically *atwrky33‐2* and *atmyb15‐1*, which exhibit reduced levels of camalexin and scopoletin, respectively. Quantitative comparisons between these genotypes confirmed the method's capacity to detect significant reductions in phytoalexin concentrations. Compared to conventional methods, our approach offers a significantly faster analysis with minimal sample consumption, requiring only 2 μL per run. Each analysis is completed in just 30 s, a stark contrast to the several minutes needed for liquid chromatography or the hours required by some gas chromatography methods. While our method is less sensitive than separation‐based techniques, which have reported linear ranges for camalexin down to 0.1 μg/g [17, 18, 20], it is highly effective for analyzing elicited *Arabidopsis* samples. Specifically, our method reliably quantifies scopoletin when concentrations exceed 0.15 μg/g and camalexin above 1.25 μg/g, as demonstrated by the linear range of the calibration curve and by the analysis of the *Arabidopsis* sample.

Phytoalexins play crucial roles in plant–pathogen interactions, and understanding their regulation is essential for enhancing plant defense strategies. By providing a reliable and fast method for quantifying these key metabolites, our work lays the groundwork for deeper insights into plant defense mechanisms against biotic and abiotic stresses.

This advancement supports future research in plant biology and biotechnology, contributing to agricultural and pharmaceutical developments. Our study highlights the significance of MALDI‐HRMS in metabolomics research and its potential for elucidating complex biological processes in plants. By offering valuable analytical methods and integrating them with biological questions, we aim to advance our understanding of plant metabolism and physiology, ultimately contributing to increased agricultural productivity and crop yields.

## Author Contributions

**Leonardo Parasecolo:** methodology, writing – original draft, writing – review and editing, formal analysis. **Ivan M. Monsalvo:** methodology, resources, writing – original draft, writing – review and editing, funding acquisition. **Nikola Kovinich:** conceptualization, funding acquisition, resources, supervision. **Demian R. Ifa:** conceptualization, supervision, resources, funding acquisition.

## Supporting information


**Figure S1** MSMS fragmentation patterns comparison between samples and standard solution.

**Figure S2** Blank matrix analysis.

**Figure S3** Blank matrix analysis.

**Figure S4** Signal‐to‐noise ratio of samples at the established LOD and LOQ.

**Table S1** Analytical replicates and ion intensity measurements of camalexin and scopoletin in different genetic lines (Columbia WT, *atwrky33‐2*, *atmyb15‐1*) under treatments with the elicitor flg22 and DMSO as control.

**Data S1** Supporting information.

## Data Availability

The data that support the findings of this study are available from the corresponding author upon reasonable request.
