# Supplementary figures and images for: Development of a Matrix‐Assisted Laser Desorption Ionization High Resolution Mass Spectrometry Method for the Quantification of Camalexin and Scopoletin in Arabidopsis thaliana
Source: Rapid Commun Mass Spectrom. 2024 Dec 18;39(6):e9973. doi: 10.1002/rcm.9973 (PMC11655771; doi:10.1002/rcm.9973)

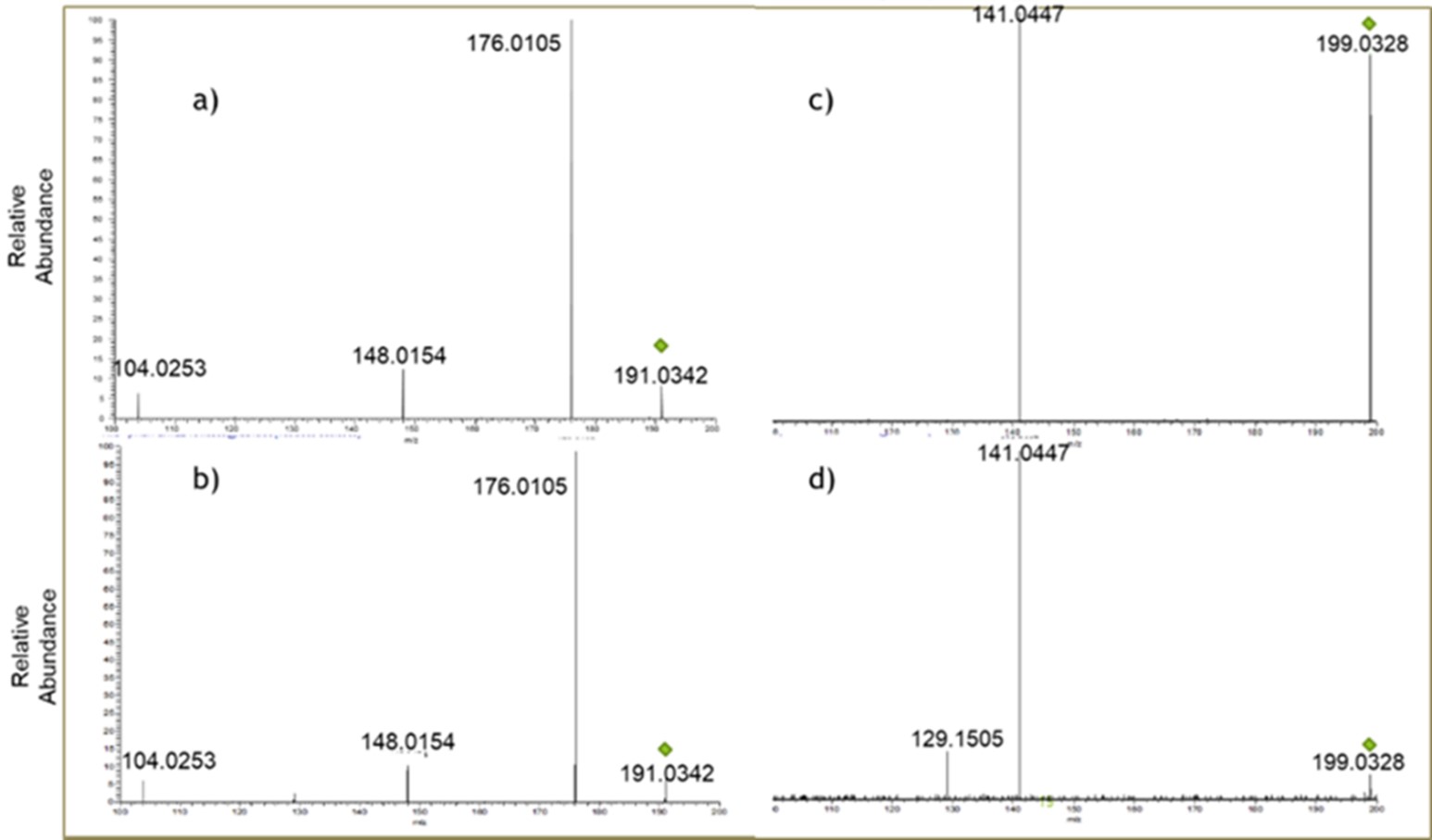

Supplement: Supplementary file 1 — Figure S1 MSMS fragmentation patterns comparison between samples and standard solution. [file RCM-39-e9973-s005.jpg]

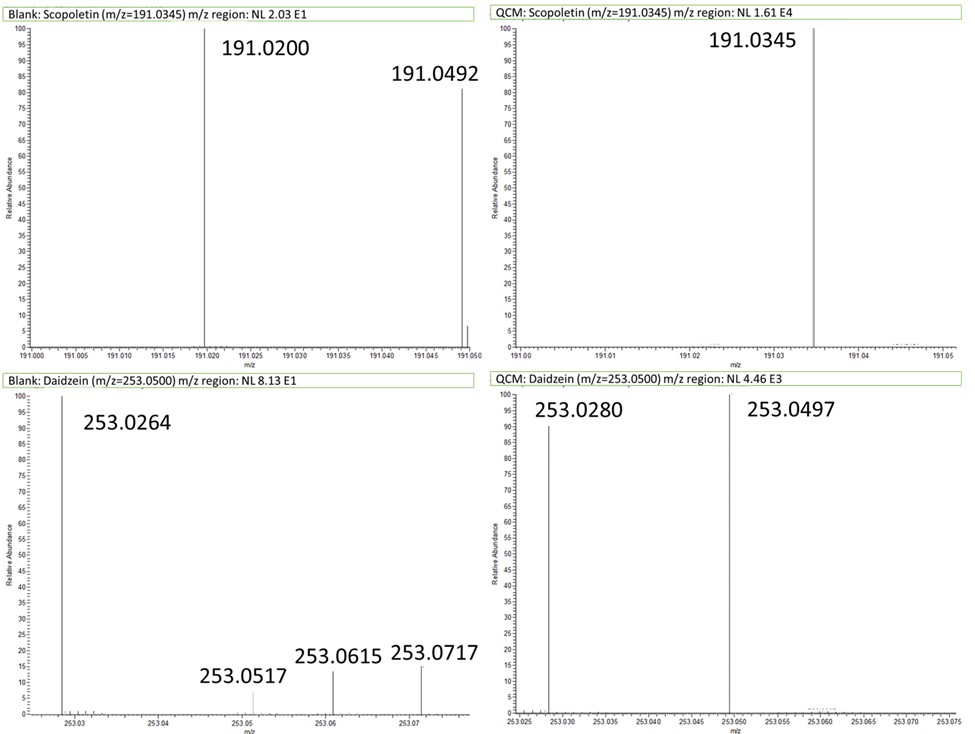

Supplement: Supplementary file 2 — Figure S2 Blank matrix analysis. [file RCM-39-e9973-s004.jpg]

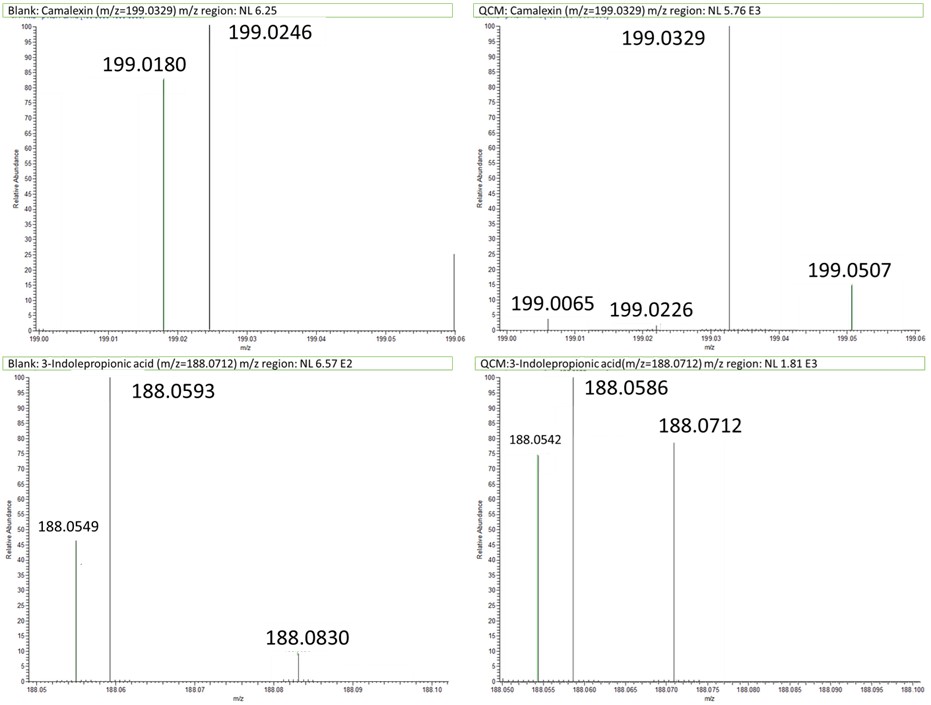

Supplement: Supplementary file 3 — Figure S3 Blank matrix analysis. [file RCM-39-e9973-s002.jpg]

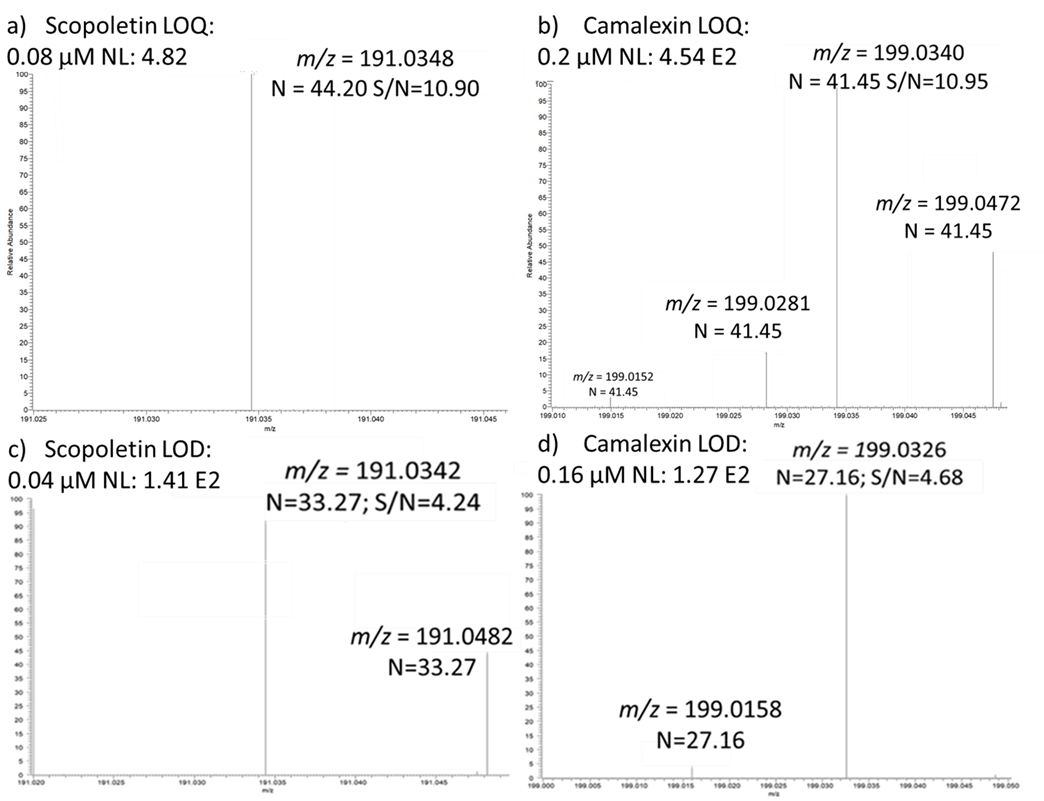

Supplement: Supplementary file 4 — Figure S4 Signal‐to‐noise ratio of samples at the established LOD and LOQ. [file RCM-39-e9973-s001.png]
