# Supplementary material for: Development of a Matrix‐Assisted Laser Desorption Ionization High Resolution Mass Spectrometry Method for the Quantification of Camalexin and Scopoletin in Arabidopsis thaliana
Source: Rapid Commun Mass Spectrom. 2024 Dec 18;39(6):e9973. doi: 10.1002/rcm.9973 (PMC11655771; doi:10.1002/rcm.9973)
Supplement: Supplementary file 6 — Data S1 Supporting information. [file RCM-39-e9973-s003.docx]

**
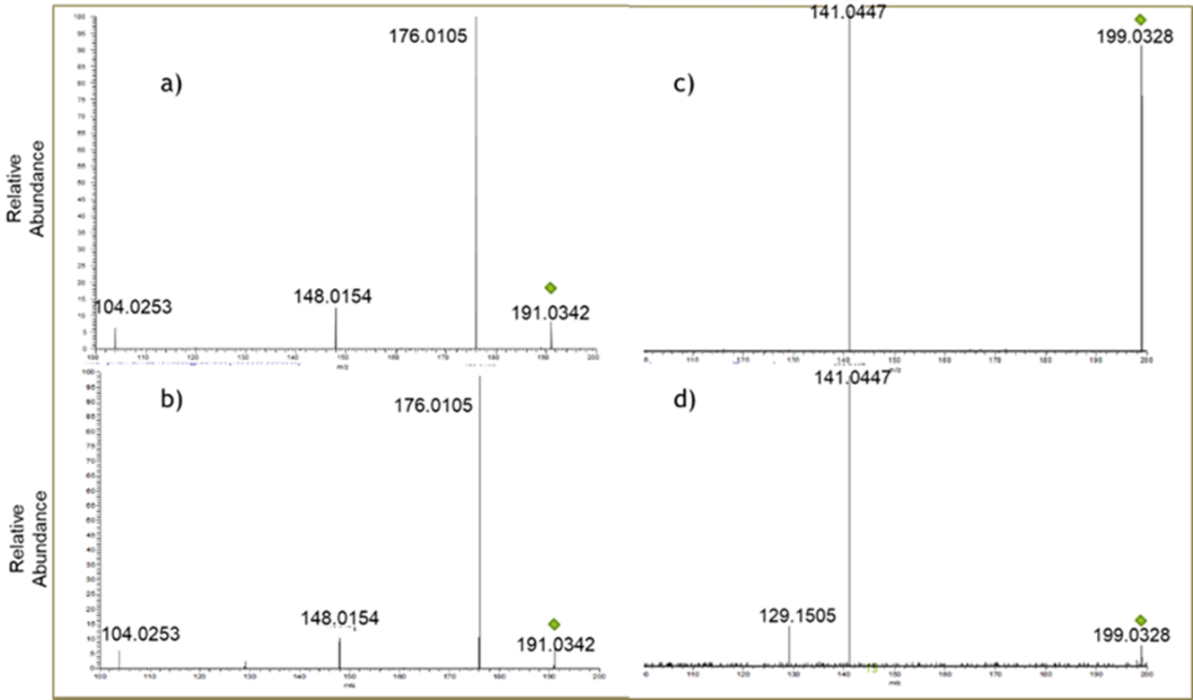
Figure S1: MSMS fragmentation patterns comparison between samples and standard solution**

Figure S1: MSMS fragmentation patterns a) Scopoletin in the standard solution, b) Scopoletin in the plant extracts, c) Camalexin in the standard solution, and d) Camalexin in the plant extract. Matching fragmentation patterns confirm compound identities.

**Figure S2 Blank matrix analysis.
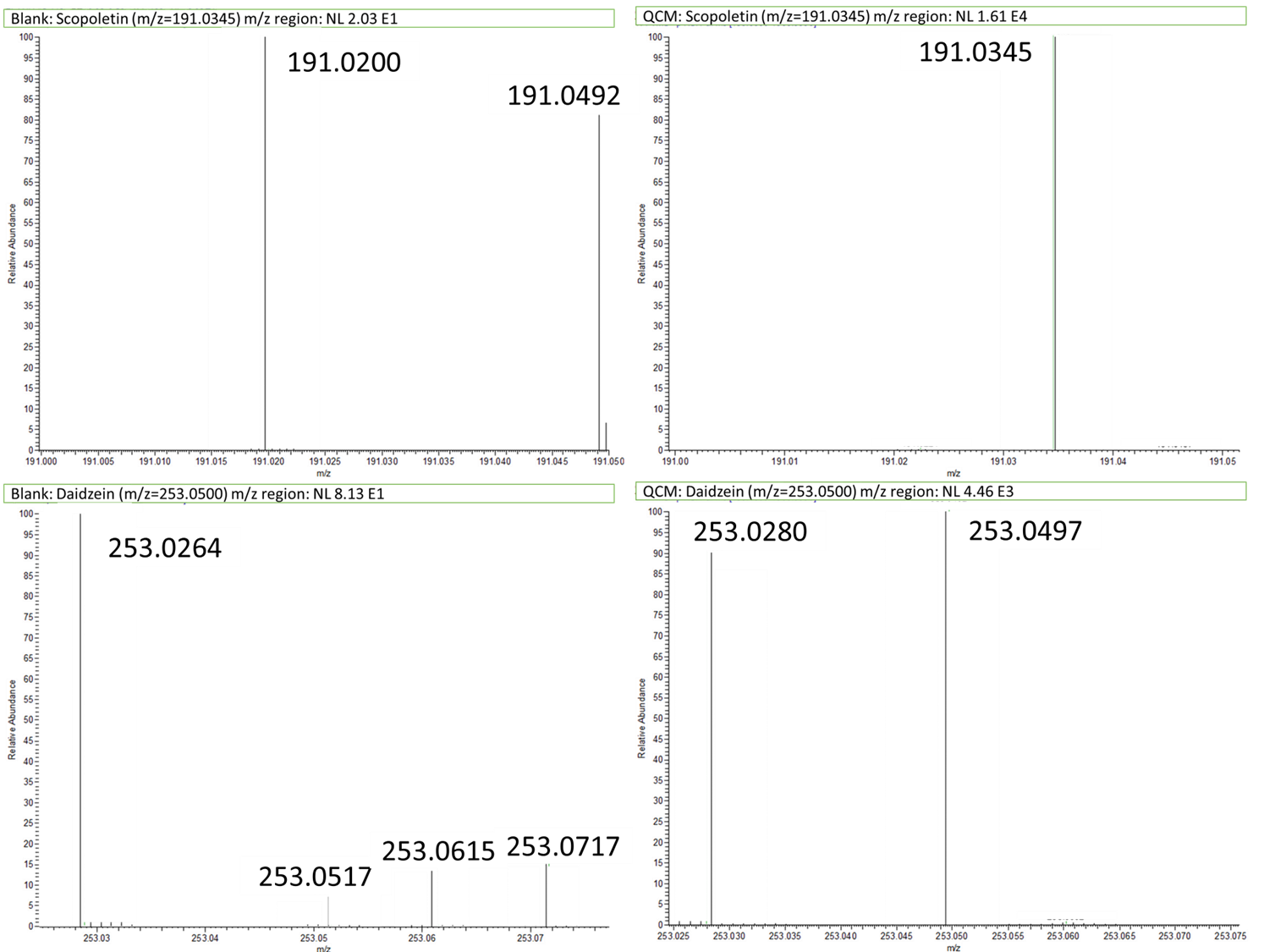
**

On the left, the mass spectrum of the blank samples; on the right, the spiked QCM samples. From top to bottom: Scopoletin (*m/z* = 191.0345), Daidzein (*m/z*=253.0500).

**Figure S3 Blank matrix analysis.**
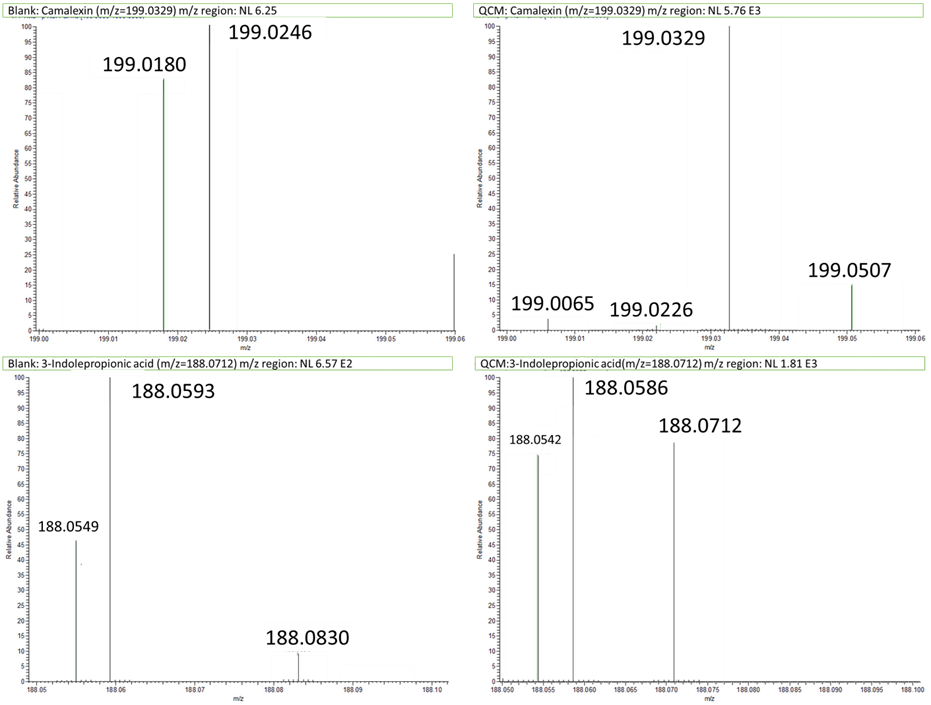


On the left, the mass spectrum of the blank samples; on the right, the spiked QCM samples. From top to bottom: Camalexin (*m/z* = 199.0329), 3-Indolepropionic acid (*m/z*=188.0712).

**Figure S4: Signal to noise ratio of samples at the established LOD and LOQ**


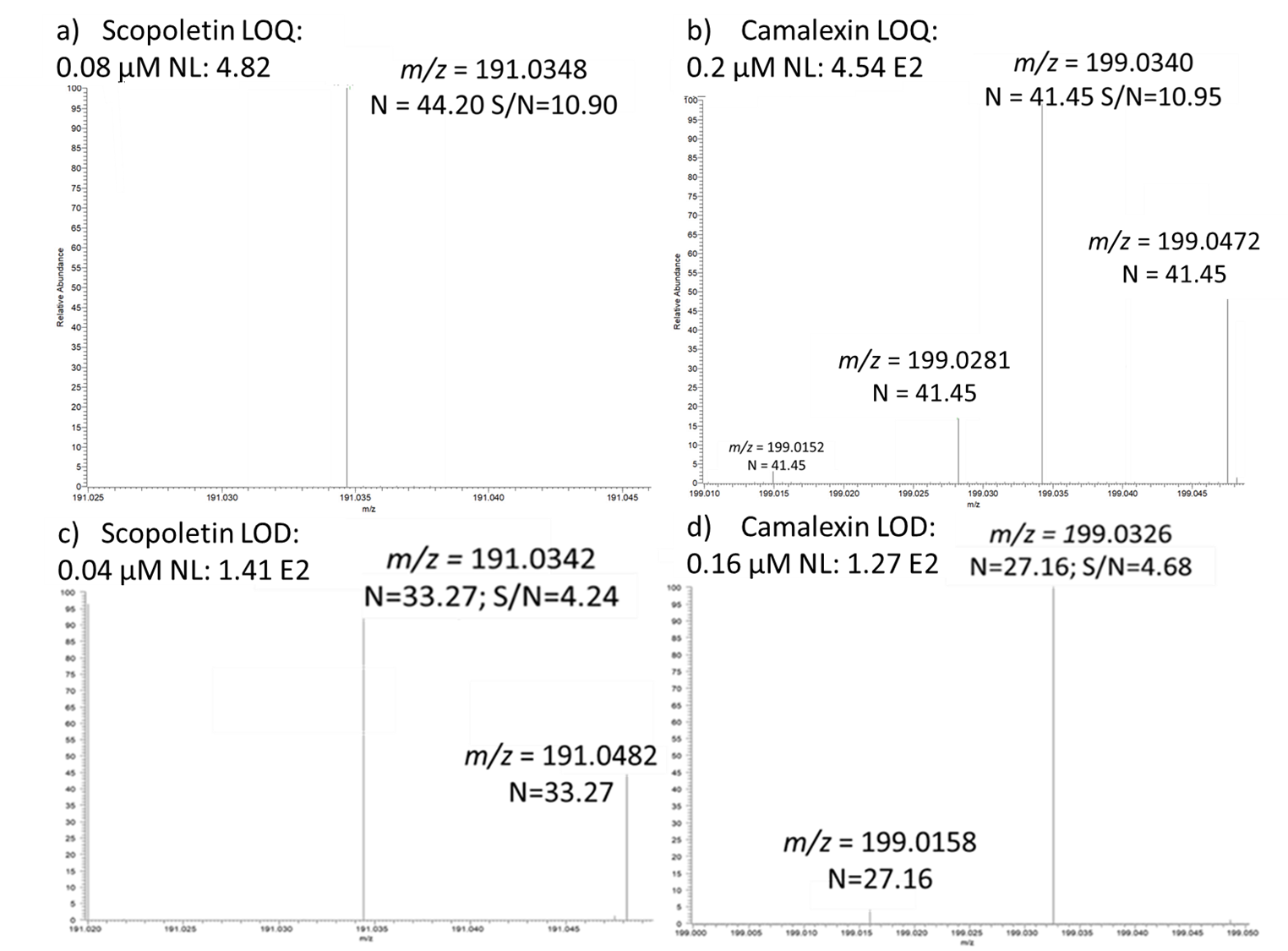


a) LOQ for scopoletin at 0.08 µM. b) LOQ for camalexin at 0.2 µM. c) LOD for Scopoletin at 0.04 µM d) LOD for camalexin at 0.16 µM. Noise (N) was estimated using the root-mean-square method over baseline regions. The S/N ratio reflects the relationship between the signal intensity of the peak of interest and the corresponding baseline noise. The S/N was calculated only for the peak of interest.

**Table S1 Analytical Replicates and Ion Intensity Measurements of Camalexin and Scopoletin in Different Genetic Lines (Columbia WT, atwrky33-2, atmyb15-1) under Treatments with the Elicitor flg22 and DMSO as Control**


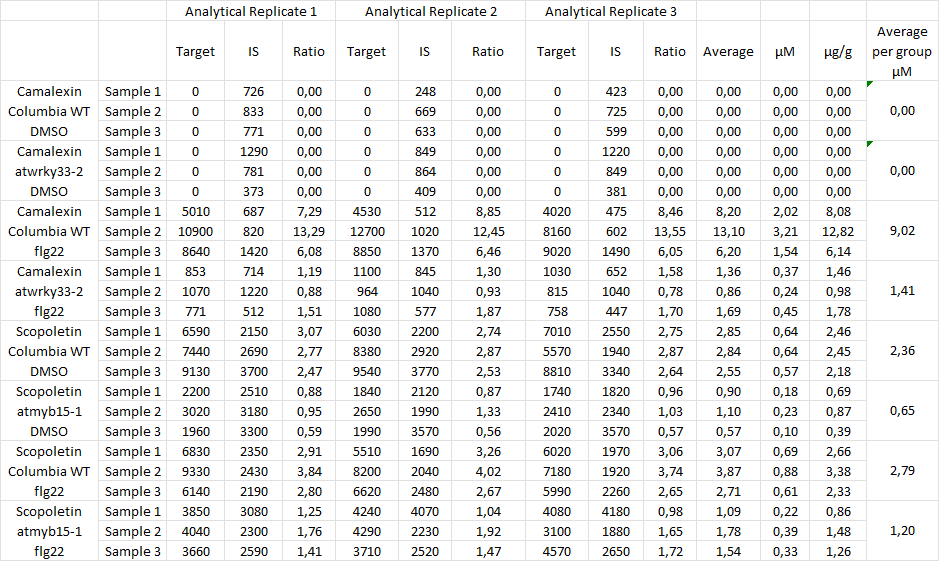


Table S1 reports ion intensities for target compounds and internal standards (IS) from each analytical replicate across different genetic lines and treatment conditions. Ratios, averages, and calculated concentrations are presented in µM and µg/g, with a final column showing the average concentration per group in µM.
